# Testosterone and androstanediol glucuronide among men in NHANES III

**DOI:** 10.1186/s12889-018-5255-6

**Published:** 2018-03-09

**Authors:** Chuan Wei Duan, Lin Xu

**Affiliations:** 1 Guangzhou Number 12 Hospital, Guangzhou, China; 2 School of Public Health, Sun Yat-sen University, 2nd Zhongshang Road, Guangzhou, Guangdong Province China

**Keywords:** Testosterone, Androstanediol glucuronide, Correlation

## Abstract

**Background:**

Most of the androgen replacement therapies were based on serum testosterone and without measurements of total androgen activities. Whether those with low testosterone also have low levels of androgen activity is largely unknown. We hence examined the association between testosterone and androstanediol glucuronide (AG), a reliable measure of androgen activity, in a nationally representative sample of US men.

**Methods:**

Cross-sectional analysis was based on 1493 men from the Third National Health and Nutrition examination Survey (NHANES III) conducted from 1988 to 1991. Serum testosterone and AG were measured by immunoassay. Kernel density was used to estimate the average density of serum AG concentrations by quartiles of testosterone.

**Results:**

Testosterone was weakly and positively correlated with AG (correlation coefficient = 0.18). The kernel density estimates show that the distributions are quite similar between the quartiles of testosterone. After adjustment for age, the distributions of AG in quartiles of testosterone did not change. The correlation between testosterone and AG was stronger in men with younger age, lower body mass index, non-smoking and good self-rated health and health status.

**Conclusions:**

Serum testosterone is weakly correlated with total androgen activities, and the correlation is even weaker for those with poor self-rated health. Our results suggest that measurement of total androgen activity in addition to testosterone is necessary in clinical practice, especially before administration of androgen replacement therapy.

## Background

Lower serum testosterone was associated with high levels of cardiovascular risk factors, such as blood pressure, dyslipidemia or atherosclerosis from observational studies [1]. However, evidence from large randomized controlled trials (RCTs) or meta-analysis of RCTs shows that treatment with testosterone in elderly men is not beneficial and even harmful [2]. Such discrepancies may be due to the common causes of low testosterone and cardiovascular disease, such as underlying illness or aging, or may be because the measurement of serum testosterone does not fully reflect the total circulating androgen exposure.

Serum testosterone as an evaluation of androgen activity is widely used for diagnosis of androgen deficiency. However, the success of using anti-androgens permitting combined androgen blockade plus castration in treating castration-resistant prostate cancer suggests that measurement of serum testosterone does not provide the whole picture of androgen activity. Moreover, an observational study showed that serum testosterone concentrations declined by 97% following castration in elderly men [3], but the total androgen metabolites has only reduced by 59% [3], which suggests that 41% of androgens remained in the prostate and free to stimulate prostate cancer after castration. Glucuronidated androgen metabolites, mainly including androsterone glucuronide (ADT-G) and androstanediol glucuronide (androstane-3α, 17β-diol-3-glucuronide and androstane- 3α, 17β-diol-17-glucuronide), reflect the total androgen metabolites [4, 5].

Androgen prescription has increased substantially in US in recent years, and among the users of androgen replacement therapy (ART), most men did not have clear evidence of androgen deficiency [6]. Moreover, while about 3 out of 4 ART users had measured testosterone levels [6], most of them did not have test on glucuronidated androgen metabolites, as such are not routine measurements in clinical practice. Whether those with low testosterone also have low levels of androgen activity is largely unknown. As result, there may be an over-diagnosis of androgen deficiency and subsequent inappropriate use of testosterone replacement therapy and inadequate response to treatment in older men. More research is needed to determine the correlation between testosterone and total androgen metabolites, such glucuronidated androgens, particular given the concerns about the adverse cardiovascular effects in older people [2]. Therefore, we hypothesized that individuals with low serum testosterone would not necessarily have low levels of glucuronidated androgens.

## Methods

### Ethics, consent and permissions

The Third National Health and Nutrition Examination Survey (NHANES III) is a cross-sectional study conducted in the US by the National Center for Health Statistics between 1988 and 1994. Details of the NHANES III have been reported elsewhere [7]. The protocols for the conduct of NHANES III were approved by the institutional review board of the National Center for Health Statistics, Centers for Disease Control and Prevention. Informed consent was obtained from all participants. The assay of stored serum specimens for the Hormone Demonstration Program was approved by the Institutional Review Boards at the Johns Hopkins Bloomberg School of Public Health and the National Center for Health Statistics, Centers for Disease Control and Prevention.

### Study variables

Of the 2205 males aged at least 12 years who participated in NHANES III phase 1 (1988–1991) and attended a morning examination session, 1637 without taking androgen replacement therapy had surplus sera, previously stored at − 70 °C, assayed for sex steroids using competitive electrochemiluminescence immunoassays on the 2010 Elecsys autoanalyzer (Roche Diagnostics, Indianapolis, IN, USA) for serum testosterone and an enzyme immunoassay (Diagnostic Systems Laboratories, Webster, TX, USA) for a glucuronidated androgen metabolite, androstanediol glucuronide (AG) in 2005 at Children’s Hospital, Boston, Massachusetts [8, 9]. The detection limits of the assays were 0.02 ng/mL and 0.33 ng/mL for testosterone and AG, respectively. The intra- and inter-assay coefficient variation ranged from 5.8% to 5.9% for total testosterone, and from 5.0% to 9.5% for AG [10].

### Statistical analysis

Analysis of variance (ANOVA) was used to analyse the serum AG or testosterone concentrations by study characteristics, giving mean, standard deviation (SD) and *p* values for the differences among subgroups. Correlation coefficient between testosterone and AG was calculated using Pearson correlation test. Kernel density was used to estimate the average density of serum AG concentrations by quartiles of testosterone. We also checked the age adjusted distributions of AG by quartiles of testosterone by weighting the US sample back to the US population and taking account of the complex survey design. As androgens may vary with age [11], adiposity, smoking and health status [12], we also checked for the correlation coefficients between testosterone and AG by these health conditions. A two sided *P*-value < 0.05 was considered to be statistically significant. All data analysis was done by STATA/IC 14.0.

## Results

Of the 1637 men attended a morning examination session and had surplus sera, 1493 were aged 18 years or older and had values for both testosterone and AG. Of these 1493 men, the mean (standard deviation (SD), range) age was 47.2 (SD = 19.3, range 18–90) years. Men who were older and had higher BMI, poor health status and self-rated health tended to have lower testosterone and AG (Table 1).

**Table 1:** Serum concentrations of 13α-Diol-G and testosterone by demographic characteristics

		Number (%)	13α-Diol-G, ng/ml, mean (SD)	Testosterone, ng/ml, mean (SD)
Age, years	< 65	1135 (76)	13.68 (8.95)	5.59 (2)
	≥65	358 (24)	10.91 (8.37)	4.34 (1.87)
	P value^‡^	–	< 0.001	< 0.001
Tertiles of BMI, kg/m^2^	1st (≤23)	475 (31.8)	12.54 (9.36)	6.18 (2.23)
	2nd (24–27)	509 (34.1)	13.27 (8.77)	5.32 (1.89)
	3rd (≥27)	509 (34.1)	13.21 (8.56)	4.43 (1.58)
	P value^‡^	–	0.36	< 0.001
Smoking	Never	536 (35.9)	13.35 (8.25)	5.27 (1.89)
	Former	489 (32.8)	12.74 (10.16)	4.52 (1.82)
	Current	468 (31.4)	12.92 (8.15)	6.1 (2.11)
	P value^‡^	–	0.53	< 0.001
Health status^a^	Good	885 (59.3)	13.55 (9.14)	5.65 (2.01)
	Poor	608 (40.7)	12.23 (8.46)	4.76 (1.96)
	P value^‡^	–	0.005	< 0.001
Self-rated health	Excellent	540 (37.2)	13.79 (9.5)	5.65 (1.98)
	Very good	326 (22.5)	12.96 (8.92)	5.39 (2.11)
	Good	440 (30.3)	12.87 (8.25)	5.11 (1.95)
	Fair	128 (8.8)	10.63 (7.68)	4.41 (1.97)
	Poor	17 (1.2)	9.8 (6.54)	3.38 (2.01)
	P value^‡^	–	0.003	< 0.001
Ethnicity	Non-Hispanic white	675 (45.2)	13.36 (9.34)	5.0 (1.93)
	Non-Hispanic black	369 (24.7)	12.55 (8.87)	5.71 (2.19)
	Mexican-American	391 (26.2)	13.27 (8.41)	5.46 (2)
	Other	58 (3.9)	10.24 (5.8)	4.8 (2)
	P value^‡^	–	0.05	< 0.001

^a^Poor health status was defined as self-reported physician diagnosis of any of the following diseases: diabetes, hypertension, high cholesterol, heart disease or stroke, or at least one hospitalization during the past 12 months

^‡^*P* values were from ANOVA test

**Fig. 1:**
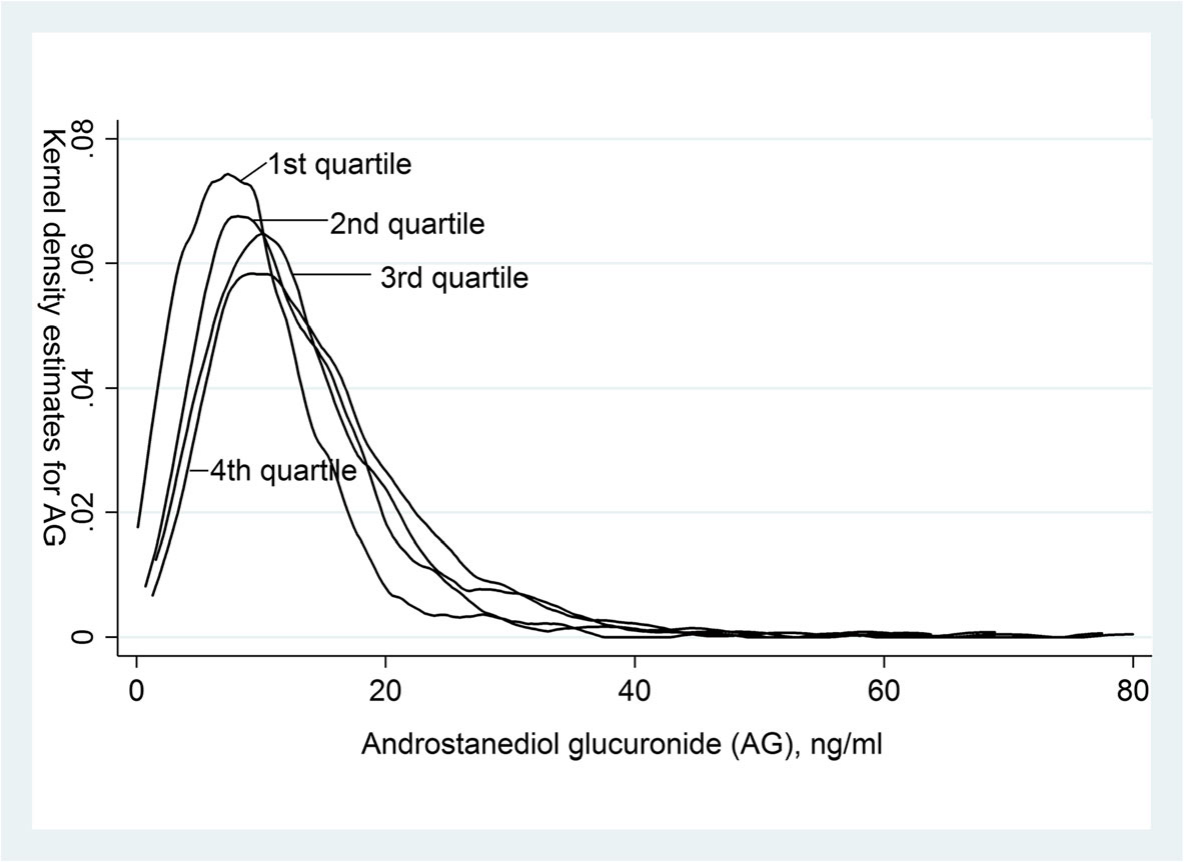
Kernel density estimates for androstanediol glucuronide by quartiles of testosterone (testosterone concentrations in the 1st to 4th quartile were ≤3.91 ng/ml; 3.92–5.11 ng/ml, 5.12–6.55 ng/ml and 6.56–13.82 ng/ml, respectively)

Testosterone was weakly and positively correlated with AG (correlation coefficient = 0.18). The kernel density estimates show that the distributions are quite similar between the quartiles of testosterone (Fig. 1). After adjustment for age, the distributions of AG in quartiles of testosterone did not change. (Figure not shown) The correlation between testosterone and AG was stronger in men with younger age, lower BMI, non-smoking, good self-rated health and health status (Table 2).

**Table 2:** Pearson correlation coefficients between 13α-Diol-G (ng/ml) and testosterone (ng/ml) by different health conditions

		Correlation coefficient	95% confidence interval	*P* value
Age, years	< 65	0.17	0.12 to 0.23	< 0.001
	≥65	0.12	0.02 to 0.22	0.03
Tertiles of BMI, kg/m^2^	1st (≤23)	0.25	0.17 to 0.34	< 0.001
	2nd (24–27)	0.17	0.08 to 0.25	< 0.001
	3rd (≥27)	0.22	0.13 to 0.30	< 0.001
Smoking	Never	0.22	0.13 to 0.30	< 0.001
	Former	0.19	0.1 to 0.27	< 0.001
	Current	0.20	0.11 to 0.28	< 0.001
Health status^a^	Good	0.24	0.12 to 0.25	< 0.001
	Poor	0.17	0.09 to 0.25	< 0.001
Self-rated health	Excellent	0.21	0.14 to 0.3	< 0.001
	Very good	0.17	0.06 to 0.27	0.002
	Good	0.12	0.03 to 0.21	0.01
	Fair	0.11	−0.07 to 0.27	0.24
	Poor	−0.22	− 0.64 to 0.28	0.37
Ethnicity	Non-Hispanic white	0.20	0.11 to 0.25	0.18
	Non-Hispanic black	0.25	0.1 to 0.29	0.20
	Mexican-American	0.28	0.13 to 0.32	0.22
	Other	0.12	−0.06 to 0.43	0.20

^a^Poor health status was defined as self-reported physician diagnosis of any of the following diseases: diabetes, hypertension, high cholesterol, heart disease or stroke, or at least one hospitalization during the past 12 months

## Discussion

Our results for the first time showed that even within the lowest quartile of testosterone, the distribution of AG was quite similar to the other quartiles of higher testosterone, suggesting a very weak correlation between serum levels of testosterone and AG. Clinical diagnosis of androgen deficiency based on measurement of serum testosterone might not be able to capture the total androgen metabolites.

Total testosterone is usually used to determine whether or not testosterone levels are normal in clinical practice, whereas levels of bio-available or free testosterone are often used to assess the underlying causes of an abnormal total testosterone level, because the relative proportions of bound and unbound testosterone may vary in different conditions. Our previous study showed that the measures of testosterone (i.e., total, free or bioavailable testosterone) have consistent associations with age and ethnicity, as the levels of free and bioavailable testosterone can be calculated using the Vermeulen’s formula [13] based on measures of total testosterone, sex hormone-binding globulin (SHBG) and albumin [14]. All these 3 measures of testosterone are highly correlated. Moreover, as the level of free testosterone is determined in part by SHBG, androgen deficiency may be misclassified in men with low SHBG [15]. Thus, more comprehensive methods of assessing total androgen metabolites (i.e., AG or androsterone glucuronide) are warranted.

The results of the present study have important clinical implications. First, our findings may partly explain the discrepancies of the association of testosterone and cardiovascular disease (CVD) between observational studies and large RCTs. Observationally, low serum testosterone has been linked to elevated blood pressure, dyslipidemia, endothelia dysfunction, atherosclerosis and incident CVD [1]. However, the most updated meta-analysis of RCTs showed a significantly harmful effect of testosterone on CVD [2]. Individuals with “low testosterone” may or may not have low total androgens. Testosterone administration in men with low testosterone but normal or high androgens may not be beneficial and could even be dangerous. Recent studies showed that serum AG, but not testosterone, was associated with unhealthy cardiovascular risk factors and ischemic heart disease [9], which was more consistent with results from large RCTs showing increasing androgens had adverse effects on cardiovascular system [16–18]. Second, there is some evidence that the association of testosterone administration with CVD vary with age, with the adverse effect being more pronounced in people aged 65 or more than those younger [19]. One of the possible explanations is that younger men tend to have both lower testosterone and androgen activities while older men with low testosterone may not necessarily have low androgen activities, as a higher correlation between testosterone and AG was found in younger men. Testosterone administration in those without low androgen activities might not provide beneficial effects. Measurement of AG in older people in addition to testosterone is thus highly recommended before administering ART. Third, low serum testosterone has been linked to some chronic diseases or frailty in observational studies [20], although the causal direction is apt to be from underlying illnesses to low testosterone rather than the reverse. Our results that lower correlation between testosterone and AG in participants with poor self-rated health or health status thus deserve further attention, as treating low serum testosterone in those with some underlying diseases by ART might increase the risk of androgen-related diseases. As prescriptions for testosterone replacement therapies based on low serum testosterone have more than doubled since 2006 to 5.6 million in 2011, and tripled to $5 billion by 2017, according to forecasts by Global Industry Analysts [21], cautious should be taken to ensure that the health benefits of testosterone therapy outweigh the potential increased risk. Therefore, it is important to use a holistic measurement of androgen activity in addition to the serum levels of testosterone, to facilitate the administration of ART.

There are several limitations in this study. First, the current study used AG as a proxy marker for total androgen activity but not included androsterone glucuronide [4, 5]. However, AG is highly correlated with ADT-G [5]. We thus would expect similar effects of ADT-G and AG on other risk factors or disease outcomes. Further studies using additional measurements of ADT-G are needed to confirm. Second, immunoassay rather than the gold-standard (i.e., mass spectrometry (MS)-based method) was used to measure AG or testosterone. Although the MS-based method provides more accurate measurements, immunoassay is considered to be generally valid and useful [22], and has been widely used in clinical practice as well as for large population-based research. Finally, the sample size of the current study is not large.

## Conclusion

This study shows a weak correlation between serum levels of testosterone and total androgen activities, and the correlation is even weaker for those with poor self-rated health. Our results suggest that measurement of total androgen activity in addition to testosterone is necessary in clinical practice, especially before administration of androgen replacement therapy.

## Abbreviations

ADT-G: Androsterone glucuronide
AG: Androstanediol glucuronide
ANOVA: Analysis of variance
ART: Androgen replacement therapy
CVD: Cardiovascular disease
MS: Mass spectrometry
NHANES III: The Third National Health and Nutrition Examination Survey
RCT: Randomized controlled trials
SD: Standard deviation
